# Trail making test B in postoperative delirium: a replication study

**DOI:** 10.1016/j.bjao.2023.100239

**Published:** 2023-11-03

**Authors:** Marinus Fislage, Insa Feinkohl, Friedrich Borchers, Maria Heinrich, Tobias Pischon, Dieuwke S. Veldhuijzen, Arjen J.C. Slooter, Claudia D. Spies, Georg Winterer, Norman Zacharias, BioCog Consortium

**Affiliations:** 1 Charité–Universitätsmedizin Berlin, Corporate Member of Freie Universität Berlin and Humboldt-Universität zu Berlin, Department of Anesthesiology and Operative Intensive Care Medicine (CCM, CVK), Berlin, Germany; 2 Witten/Herdecke University, Faculty of Health/School of Medicine, Witten, Germany; 3 Max-Delbrueck-Center for Molecular Medicine in the Helmholtz Association (MDC), Molecular Epidemiology Research Group, Berlin, Germany; 4 Berlin Institute of Health at Charité - Universitätsmedizin Berlin, Berlin, Germany; 5 Max-Delbrueck-Center for Molecular Medicine in the Helmholtz Association (MDC), Biobank Technology Platform, Berlin, Germany; 6 Berlin Institute of Health at Charité - Universitätsmedizin Berlin, Core Facility Biobank, Berlin, Germany; 7 Health, Medical and Neuropsychology Unit, Leiden University, Leiden, the Netherlands; 8 Leiden Institute for Brain and Cognition, Leiden, the Netherlands; 9 Department of Psychiatry, University Medical Center Utrecht, Utrecht University, Utrecht, the Netherlands; 10 Department of Intensive Care, University Medical Center Utrecht, Utrecht University, Utrecht, the Netherlands; 11 UMC Utrecht Brain Center, University Medical Center Utrecht, Utrecht University, Utrecht, the Netherlands; 12 Department of Neurology, UZ Brussel and Vrije Universiteit Brussel, Brussels, Belgium; 13 Pharmaimage Biomarker Solutions GmbH, Berlin, Germany; 14 Department of Neurology, National Taiwan University Hospital, Taipei, China

**Keywords:** Geriatric anaesthesia, Open science, Perioperative medicine, Postoperative delirium, Replication, Risk prediction, Trail making test

## Abstract

**Background:**

The Trail Making Test B (TMT-B) is indicative of cognitive flexibility and several other cognitive domains. Previous studies suggest that it might be associated with the risk of developing postoperative delirium, but evidence is limited and conflicting. We therefore aimed to replicate the association of preoperative TMT-B results with postoperative delirium.

**Methods:**

We included older adults (≥65 yr) scheduled for major surgery and without signs of dementia to participate in this binational two-centre longitudinal observational cohort study. Presurgical TMT-B scores were obtained. Delirium was assessed twice daily using validated instruments. Logistic regression was applied and the area under the receiver operating characteristic curve calculated to determine the predictive performance of TMT-B. We subsequently included covariates used in previous studies for consecutive sensitivity analyses. We further analysed the impact of outliers, missing or impaired data.

**Results:**

Data from 841 patients were included and of those, 151 (18%) developed postoperative delirium. TMT-B scores were statistically significantly associated with the incidence of postoperative delirium {odds ratio per 10-s increment 1.06 (95% confidence interval [CI] 1.02–1.09), *P*=0.001}. The area under the receiver operating characteristic curve was 0.60 ([95% CI 0.55–0.64], *P*<0.001). The association persisted after removing 21 outliers (1.07 [95% CI 1.03–1.07], *P*<0.001). Impaired or missing TMT-B data (*n*=88) were also associated with postoperative delirium (odds ratio 2.74 [95% CI 1.71–4.35], *P*<0.001).

**Conclusions:**

The TMT-B was associated with postoperative delirium, but its predictive performance as a stand-alone test was low. The TMT-B alone is not suitable to predict delirium in a clinical setting.

**Clinical trial registration:**

NCT02265263. (https://clinicaltrials.gov/ct2/show/results/NCT02265263).

Postoperative delirium (POD) remains one of the most frequent complications after surgery, especially in older adults.^1^ Episodes of postoperative delirium occurring days after surgery are linked to an increased morbidity, a higher rate of institutionalisation, increased healthcare costs, and the progression of dementia.^2^ Delirium may be preventable in some cases.^3^ To be able to assign preventive measures to patients at risk in a timely way, prediction algorithms have been suggested, but none has been adopted into clinical practice yet.^4^ Although recent studies have presented machine learning approaches as a promising new prediction tool,^5^ an easy to administer, cheap, and simple test is still a much needed option. Among a variety of widely acknowledged factors, preexisting cognitive impairment before surgery was identified to be a key risk factor of postoperative delirium.^1^ The Trail Making Test B (TMT-B) is a relatively easy to administer test which requires task switching and uses cognitive flexibility as a core dimension of executive function, guided by frontal lobe functioning.^6^, ^7^ High inter-rater reliability has also been reported.^6^ Previous studies have suggested that its preoperative performance might be associated with the risk of developing postoperative delirium.^8^, ^9^ The TMT-B as a predictor tool for postoperative delirium could potentially replace extensive preoperative diagnostic procedures, or could be used in regions of the world where healthcare institutions are short-staffed and technical devices such as imaging or laboratory equipment are not reliably available.

Four previous studies examined the relation between TMT-B performance and the incidence of postoperative delirium. Three of these studies observed an association,^8^, ^9^, ^10^ whereas one study did not report an association.^11^ Moreover, two of the studies also reported a reliable discriminatory performance using regression models including the TMT-B.^9^, ^10^ Accordingly, preoperative TMT-B results could potentially fulfil the criteria of a simple prediction tool.

Besides conflicting results, the previous studies vary in sample size, postoperative delirium assessment, statistical methods, and the curation of TMT-B data. Furthermore, none of these studies has changed clinical practice. Hence, a replication study would further evaluate a promising prediction tool and strengthen the credibility of previous findings. The BioCog study is a large framework investigating perioperative neurocognitive disorders and is specifically suited to replicate previous studies within the field. In terms of sample size, delirium screening, and overall study design, the properties of this prospective, large-scale two-centre, longitudinal observation study, can compensate for most methodological shortcomings of the previous studies.

We aim to replicate the association of preoperative TMT-B with postoperative delirium. None of the studies to be replicated investigated the predictive performance of the TMT-B alone. Thus, we further sought to evaluate the predictive properties of the TMT-B as a stand-alone test. These study findings will provide important insights into the potential use of the TMT-B as a screening tool for postoperative delirium in clinical routine.

## Methods

This article has been written in adherence with the ‘Strengthening the Reporting of Observational Studies in Epidemiology’ (STROBE) guidelines.^12^

### Replication approach

We were following a recent definition of a replication study^13^^,^^14^ in which the formerly used concepts of direct and indirect replications are replaced.^15^ Instead, a broader approach is proposed where a replication study must fulfil two criteria. Firstly, a negative result of the replication study should have the potential to put the initial results into question. Secondly, a positive result would increase confidence in the replicated studies and their findings. According to this definition, a replication study requires a strong theoretical framework rather than a completely congruent methodology. This replication approach was successfully implemented in psychology and cancer research.^16^^,^^17^ We have assembled the key characteristics of the studies to be replicated and of this replication study in Table 1.

**Table 1:** Studies in comparison. AUROC, area under the receiver operating characteristic curve; CABG, coronary artery bypass graft; CAM, Confusion Assessment Method; CI, confidence interval; CERAD, Consortium to Establish a Registry for Alzheimer's Disease; HVLT, Hopkins Verbal Learning Test; LASSO, least absolute shrinkage and selection operator; MMSE, Mini Mental State Examination; POD, postoperative delirium; sd, standard deviation; TMT, Trail Making Test. **---**not stated in the paper; **∗**median (inter-quartile range); ^†^covariates: age, Charlson Comorbidity Index, FRAIL Scale, Geriatric Depression Scale, Trail Making Test B, Word list recall, Boston Naming Test; ^‡^covariates: age, comorbidities, depression, executive function.

	Rudolph and colleagues^11^	Greene and colleagues^8^	Lindroth and colleagues^9^	Mychajliw and colleagues^10^	BioCog replication
Year of publication	2006	2009	2019	2021	2022
Sample size	80	100	97	807	841
Recruitment	Scheduled for CABG surgery	Scheduled for major, elective, noncardiac surgery	Noncardiac surgery	Participants of long-term study on neurodegeneration	Scheduled for major elective surgery
Mean age (sd)	---	64.6 (7.7)	71.7 (4.55)	---	72 (8)∗
=No delirium	73.5 (5.9)	64.4 (7.2)	71.1 (4.8)	62.9 (6.4)	71 (7)∗
=Delirium	75.7 (6.3)	66.3 (9.7)	71.6 (4.1)	66.1 (6.6)	73 (6)∗
Female (%)	---	9	45	---	41.9
=No delirium	12.5	---	41	46.6	41.0
=Delirium	32.5	---	48	53.4	45.7
Cognitive tests	HVLT (retention, recognition discrimination, learning), TMT-B, days of the weak + months of the year backwards, category fluency, Digit Span backwards, verbal fluency	TMT-A, TMT-B, Digit Symbol, substitution subtest, Wechsler adult Intelligence Scale-III (symbol search subtest)	TMT-A, TMT-B	CERAD battery, TMT-B, word list/figure recall, semantic/phonematic fluency, Boston naming test, figure copying	TMT-B
**Surgery**					
Surgery type	Coronary artery bypass graft surgery	Vascular, urology, general, thoracic, orthopaedic	Vascular, urology, general, spine	---	Any major surgery >60 min
Anaesthaesiological handling	---	---	---	---	Intraoperative electroencephalography
**POD assessment**					
Begin	Day 2 after surgery	---	Day 1 after surgery	Retrospectively	Day of surgery
Interval	Once daily	Once daily	Twice daily	n.a.	Twice daily
End	Not stated	Day 3 after surgery	Day 4 after surgery	n.a.	Day 7 after surgery
Tests	CAM/CAM-ICU, MMSE, delirium symptom interview, Memorial Delirium Assessment Scale, Digit Span	CAM	CAM, 3D-CAM, Delirium Rating Scale-R-98 (DRS)	Questionnaire covering possible delirium symptoms over a period of 8 yr	Nursing Delirium Screening Scale (Nu-DESC), Richmond Agitation Sedation Scale (RASS), CAM, CAM-ICU, and chart review
POD incidence (%)	50	16	32	7.2	18
**Trail Making Test B (TMT-B)**					
Time (s)—mean (sd)	---	135.0 (75.5)	98.7 (52.4)	---	103.9 (57)∗
=No delirium	---	119.3 (63.8)	89.92 (46.7)	---	101.0 (52)∗
=Delirium	---	217.8 (79.9)	117.48 (60.0)	---	118.0 (59)∗
Transformation of TMT-B estimate	Categorising in 0.5 sd below/above mean	None	None	z-standardisation	None
TMT testing	---	---	---	---	According to test manual
Curation of TMT data	---	---	---	---	Plausibility by two assessors
Covariate	Age, sex, education (<high school, high school, >high school), high comorbidity (Charlson Comorbidity Index ≥3)	Geriatric Depression Scale—short form, Digit Symbol Test, Symbol Search Test	National Surgical Quality Improvement Program – Serious Complications (NSQIP-SC)	Age, sex, comorbidities, depression, frailty, and the sum of taken drugs	None
Justification	---	Results of univariable analysis	LASSO	---	Assessing TMT-B as stand-alone test
Statistical analysis	Poisson regression	Logistic regression	Logistic regression	Logistic regression	Logistic regression
Association with POD	None	Yes	Yes	Yes	Yes
Estimate	Hazard ratio	Odds ratio	Odds ratio	Odds ratio	Odds ratio
Effect size	1.05 (CI 0.79–1.38)	1.02 (CI 1.01–1.04)	---	0.75 per sd (CI 0.57–0.98)^†^	1.006 per second (CI 1.002–1.009)
AUROC	---	---	0.81 (CI 0.72–0.90)	0.74 (CI 0.68–0.81)^‡^	0.60 (CI 0.55–0.64)
Outlier definition	None	None	None	None	ROUT Q=1%
Missing data	---	---	Little’s test Multiple imputations sensitivity analysis	---	Complete case analysis Sensitivity analysis with missing data
Comments	TMT-B not main focus, only part of a composite score among a variety of other tests	Sample size for preliminary analysis; 91% male subjects	Study focuses on delirium severity; incidence was exploratory outcome	Different types of delirium assessed, 69% of cases were attributed to surgery, inconsistent use of covariates	

### Study setting ansd study population

The data for this replication study were derived from a multicentre prospective observational cohort study. The ‘Biomarker Development for Postoperative Cognitive Impairment in the Elderly’ study (BioCog; www.biocog.eu) aimed to develop reliable biomarkers for perioperative neurocognitive disorders.^18^ The study was funded by the European Union and was registered (NCT02265263).

The study design was consistent with the regulations of the responsible ethics committees (No. EA2/092/14 in Berlin, Germany and No. 14–469 in Utrecht, Netherlands). To participate, patients were required to provide written informed consent. Starting in October 2014, patients were enrolled until September 2019 at both study centres. Study staff invited patients to participate in the study before their preoperative consultation with an anaesthesiologist.

For inclusion, the study protocol required patients to be 65 yr or older and score >23 points in the Mini-Mental State Examination. Participants needed to be scheduled for elective surgery with an anticipated duration of anaesthesia exceeding 60 min. Exclusion criteria comprised severe and uncompensated visual or hearing disturbances, psychiatric or neurological diseases, or psychotropic medication or any other condition interfering with cognitive testing ability (e.g. restriction of motion) (https://clinicaltrials.gov/ct2/show/NCT02265263).

### Outcome

The occurrence of postoperative delirium served as the outcome variable. Postoperative delirium was defined in accordance with the Diagnostic and Statistical Manual of Mental Disorders (DSM) 5 and current guidelines.^19^ To depict the fluctuating character of postoperative delirium, patients were screened twice a day until discharge or until day 7 after surgery. During blended learning and bench-to-bedside courses, study physicians instructed doctoral students, study nurses, and study assistants to perform the delirium assessment. To further ensure the reliability of delirium assessments, a standard operating procedure was developed.

We examined patients’ delirium status by applying the Nursing Delirium Screening Scale (Nu-DESC), Confusion Assessment Method (CAM), Confusion Assessment Method for the Intensive Care Unit score (CAM-ICU), and structured chart review. Delirium screening was performed along with the assessment of sedation and pain including the Richmond Agitation and Sedation Scale (RASS), Numeric Rating Scale (NRS), and Behavioural Pain Scale (BPS/BPS-NI). The criteria to diagnose postoperative delirium were predefined and required one of the following test results: i) ≥2 cumulative points on the Nu-DESC, ii) a positive CAM score, or both, iii) a positive CAM-ICU score or iv) evidence of delirium by chart review.

### Trail Making Test B

A pen-and-paper version of the TMT was conducted according to the prespecified instructions of the test manual.^20^ This includes administration of both parts, A and B. For this replication study, we solely used the presurgical data of TMT part B.

The TMT-B requires participants to use their dominant hand to draw a line alternating between numbers in a numerical order and letters in an alphabetical order (1-A-2-B-3, etc.). The test result is the time needed to accomplish the task, which is a continuous score in seconds. The longer the time needed to finish the TMT, the greater the difficulty a participant has with cognitive flexibility. Testing was carried out by doctoral students and study nurses, who underwent specific training to administer the task. To this end, two neuropsychologists developed a standard operating procedure. We documented and later evaluated the accompanying circumstances of each test session. In adherence with the test manual,^20^ the total test time was a maximum of 300 s (5 min) for TMT part B. The stability of cognitive data was confirmed by using the data of a control group that did not undergo surgery.^21^ This non-surgical control group consisted of 45 participants, who underwent the BioCog cognitive test battery at baseline, after 7 days and after 3 months. All tests showed good to excellent test–retest reliability, including the TMT-B.

Two assessors independently examined the test protocols and gave recommendations for the further use of data. When patients exceeded a 300 s (5 min) threshold, testing was considered to be impaired. When patients were unable to adequately participate, TMT-B results were also classified impaired or even ineligible for analysis. This could be because of confusion, refusal, or the lack of aids such as glasses, bandages or any other reason for indisposition. If the testing was not conducted, it was considered missing data. Impaired and missing cases have been collected and were independently analysed.

### Sample size

The sample size was specified for the BioCog study.^18^ However, the sample size for this replication study was further determined by the number of patients with available data on the TMT-B and delirium screening results. As recommended for replication studies, the sample size exceeded those of the replicated studies (Table 1).^22^

### Statistical analysis

A statistical analysis plan was predefined before analysis was conducted, but after data acquisition was finished. The alpha-level was, by convention, *P*<0.05.

For our primary analysis, we performed a simple logistic regression. Postoperative delirium (binary) was used as the dependent variable, while the required time to finish the TMT-B (in seconds) served as the independent variable. We obtained the odds ratio (OR) per increase in seconds needed to finish the TMT-B. We wanted to keep the model as simple as possible to mimic and assess its use in clinical practice. We further compared the diagnostic test accuracy of the variables age and sex *vs* TMT-B. Then we used these three variables combined. This would allow us to determine whether the TMT-B offers any advantage over data that are always available in a perioperative setting, such as age and sex. To account for patient- and surgery-related factors, we have added the type of surgery, the Charlson Comorbidity Index, and the type of anaesthesia to another adjusted logistic regression model.

To assess the diagnostic test accuracy of the TMT-B, we calculated an area under the receiver operating characteristic curve (AUROC). A minimum value of 0.7 was defined as sufficient discriminatory performance. If the AUROC exceeded a value of 0.7 we calculated a cut-off value using Youden’s index. We applied a Hosmer–Lemeshow test, to roughly estimate the calibration of the logistic regression model.

We used Graphpad Prism (version 9.5) for the statistical analyses and for the creation of graphs.

### Sensitivity and additional analyses

To undertake several sensitivity analyses, we performed two logistic regression analyses with sets of covariates, which were previously used in the replicated papers (Table 1). Variance inflating factors (VIF) helped to detect potential multicollinearity among each set of covariates. Presence of multicollinearity was assumed in case any covariate’s VIF exceeded 2.5. Missing data on covariates were considered to be missing at random. Hence, a complete case analysis was deemed appropriate. Mean imputation was applied to account for missing results of the Geriatric Depression Scale (GDS).

Firstly, Rudolph and colleagues^11^ adjusted for age, sex, education (<high school, high school, >high school), and high comorbidity (Charlson Comorbidity Index ≥3). Corresponding variables within the BioCog study were age, sex, education (International Standard Classification of Education condensed into three categories), and Charlson Comorbidity Index. Secondly, Mychajliw and colleagues^10^ used age, sex, depressive symptoms, comorbidities, frailty, and the sum of routinely taken drugs as adjustment variables. BioCog variables, carrying comparable information, were age, sex, imputed GDS scores, Charlson Comorbidity Index, frailty (prefrail, frail, robust), and sum of taken drugs. During the analysis stage, we decided on running another logistic regression by omitting frailty as a covariate for this model because of a large number of missing values.

Greene and colleagues^8^ set up a model comprising TMT-B, GDS—short form, the Digit Symbol Test, and the Symbol Search Test. As we did not use the Digital Symbol or the Symbol Search Test for cognitive assessment, we could not use this set of covariates for a sensitivity analysis.

Lindroth and colleagues^9^ incorporated the National Surgical Quality Improvement Program – Serious Complications (NSQIP-SC) score to their prediction model. This variable was unavailable for our study sample and, therefore, we were unable to test the performance of this model.

To minimise the impact of extreme data points, we ran an additional logistic regression, for which we excluded potential outliers from our primary analysis. We used GrapPad’s ROUT tool with Q=1% to detect outliers.^23^

There are data missing on our predictive variable the TMT-B, and on our outcome, postoperative delirium. We decided on systematically studying the impact of missing or impaired TMT-B values, as we cannot rule out that those missing data points might obfuscate our primary analysis. We aimed to identify whether missing or impaired TMT-B results were associated with postoperative delirium incidence. For this logistic regression, a binary variable served as predictor, combining missing and impaired data. As we found an association of impaired and missing TMT-B with postoperative delirium, we decided to undertake a *post hoc* worst-case imputation for impaired test performances. In accordance with the test manual, we used a TMT-B result of 301 for cases that were classified impaired and added the respective cases to the primary analysis.

We further tested a suggested cut-off for the TMT-B. Based on a prior study, Greene and colleagues^8^ applied a cut-off of 154 s for finalising the TMT-B. We calculated diagnostic test sensitivity and specificity for both approaches.

## Results

**Fig 1:**
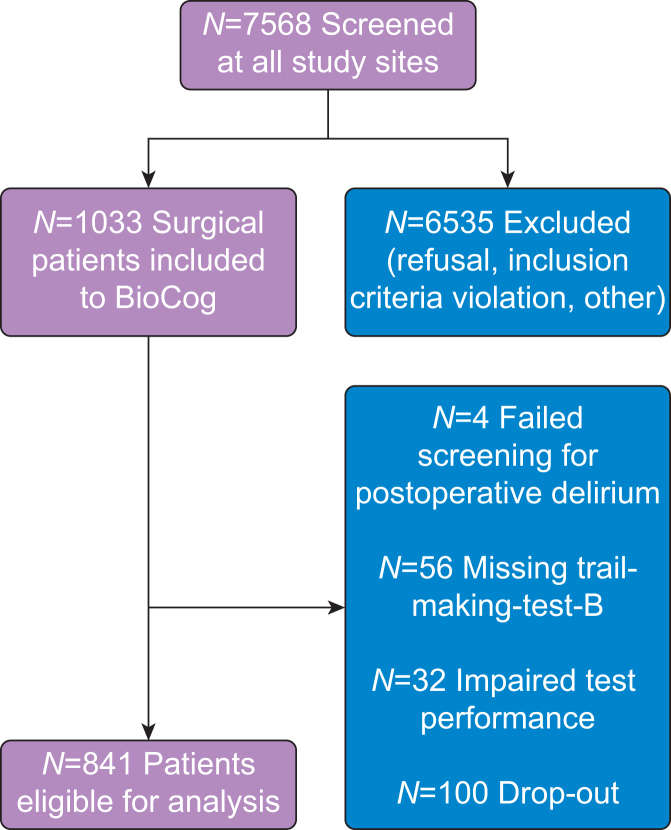
Flow chart of patient inclusion. The flow chart presents the steps from screening to study inclusion, while listing reasons for exclusion.

Of 933 surgical patients participating in the BioCog study, 32 had impaired TMT-B results and the data of 56 patients were missing. Consequently, 845 patients had valid TMT-B scores. In four cases there was no information on delirium status available (Fig. 1). The final dataset consisted of 841 patients eligible for this analysis and delirium was detected in 151 patients (18.0%). The median age of patients was 72 years (inter-quartile range [IQR] 8; 25th/75th percentile: 68/76), 71 (IQR 7; 25th/75th percentile: 68/75) for patients without and 73 (IQR 6; 25th/75th percentile: 70/76) for patients with delirium (Table 2). The median TMT-B score was 103.9 s (IQR 57; 25th/75th percentile: 80.0/137.0). Patients without delirium finished the test in a median of 101.0 s (IQR 52; 25th/75th percentile: 79.5/131.3), whereas it took those who developed delirium a median of 118.0 s (IQR 59; 25th/75th percentile: 92/151). For a summary of missing data, please see Supplementary Table S1.

**Table 2:** Patient and clinical characteristics. The table shows characteristics of all patients, those without and with postoperative delirium. For categorial variables numbers and percentages. Percentages refer to the proportion of the corresponding group (column). The *N* of patients with available data was added in bold to items with cases of missing data. ASA score, American Society of Anesthesiologists’ physical status classification; ISCED, International Standard Classification of Education; IQR, inter-quartile range (25th to 75th percentile); sd, standard deviation.

	All *N*=841	No postoperative delirium *N*=690	Postoperative delirium *N*=151
Age (yr) – median (IQR)	72 (8)	71 (7)	73 (6)
Female, *n* (%)	352 (41.9)	283 (41.0)	69 (45.7)
Study Centre Berlin, *n* (%)	600 (71.3)	490 (71)	110 (72.8)
Postoperative delirium, *n* (%)	151 (18)	---	---
Mini-Mental State Examination (MMSE)—median (IQR)	29 (2)	29 (2)	28 (3)
**Education**, ***n*****(%)**			
ISCED 1/2	131 (15.6)	110 (15.9)	21 (13.9)
ISCED 3/4	313 (37.2)	256 (37.1)	57 (37.7)
ISCED 5/6	320 (38.0)	261 (37.8)	59 (39.1)
Unknown	77 (9.2)	63 (9.1)	14 (9.3)
Time Trail-Making-Test B (s)—median (IQR)	103.9 (57)	101.0 (52)	118.0 (59)
Body mass index (BMI)—mean (sd)	27.1 (4.5) ***N*=839**	27.1 (4.4) *N*=689	27.2 (5.2) *N*=150
Sum of drugs taken per day—median (IQR)	4 (4) *N*=750	4 (4) *N*=617	5 (6) *N*=133
Charlson comorbidity index—median (IQR)	1 (2)	1 (2)	2 (2)
Diabetes, *n* (%)	175 (20.8)	138 (20.0)	37 (24.5)
Benzodiazepine premedication, *n* (%)	100 (11.9)	77 (11.2)	23 (15.2)
Duration of anaesthesia (min)—median (IQR)	205 (182.5) ***N*=826**	185 (160) *N*=675	306.0 (255.0)
**Type of anaesthesia**, ***n*****(%)**			
General	619 (73.6)	519 (75.2)	100 (66.2)
Regional	53 (6.3)	50 (7.2)	3 (2.0)
Combined	153 (18.2)	108 (15.7)	45 (29.8)
**Type of surgery**, ***n*****(%)**			
Musculoskeletal	282 (33.5)	250 (36.2)	32 (21.2)
Gastrointestinal	120 (14.3)	76 (11.0)	44 (29.1)
Cardiovascular or thoracic	89 (10.6)	62 (9.0)	27 (17.9)
Genitourinary	179 (21.3)	152 (22.0)	27 (17.9)
Otorhinolaryngology	42 (5.0)	41 (5.9)	1 (0.7)
Oral and maxillofacial	47 (5.6)	41 (5.9)	6 (4.0)
Ophthalmology	24 (2.9)	23 (3.3)	1 (0.7)
Neurosurgery	12 (1.4)	8 (1.2)	4 (2.6)
Vascular	13 (1.5)	13 (1.9)	0
Endocrine	6 (0.7)	4 (0.6)	2 (1.3)
Breast	9 (1.1)	7 (1.0)	2 (1.3)
Other	10 (1.2)	8 (1.2)	2 (1.3)
Unknown	8 (1.0)	5 (0.7)	3 (2.0)
**ASA score**, ***n*****(%)**			
ASA 1	35 (4.2)	32 (4.6)	3 (2.0)
ASA 2	514 (61.1)	447 (64.8)	67 (44.4)
ASA 3	291 (34.6)	210 (30.4)	81 (53.6)
ASA 4	1 (0.1)	1 (0.1)	---
Length of hospital stay (days)—median (IQR)	6 (6)	5 (5)	10 (13.8)
Inhouse mortality, *n* (%)	14 (1.8)	5 (0.7)	9 (6.0)

The patients’ TMT-B scores (in seconds) were statistically significantly associated with postoperative delirium incidence {OR per 10-s increment 1.06 (95% [CI] 1.02–1.01), *P*=0.001}. The AUROC was 0.60 ([95% CI 0.55–0.64], *P*<0.001) (Table 3 and Supplementary Fig. S1). Accordingly, a cut-off value was not developed. The intercept of the logistic regression model showed an estimate of −2.20 (standard error 0.23 [95% CI −2.66 to −1.75], *P*<0.001). The *P*-value for the Hosmer–Lemeshow test was *P*=0.26. When adding age and sex as covariates, the OR for the TMT-B per 10-s increment was 1.04 ([95% CI 1.01–1.08], *P*=0.02) and the AUROC was 0.63 ([95% CI 0.58–0.67], *P*<0.001). In comparison, the AUROC of age and sex alone was 0.60 ([95% CI 0.56–0.65], *P*<0.001). The association of the TMT-B with postoperative delirium was still present after correcting for factors related to the surgical procedure and the patients’ comorbidities (Table 3).

**Table 3:** Odds ratio and AUROC for Trail Making Test B with postoperative delirium. In the presented logistic regression analyses, the Trail Making Test B (TMT-B) was associated with postoperative delirium as the outcome. Apart from the primary analysis (unadjusted), there were two sensitivity analyses using covariates of replicated studies. The table displays odds ratios per 10-s increment in the TMT-B and the AUROC alongside their respective 95% CI. Unadjusted: *N*=841. Adjusted 1: covariates were age and sex; *N*=841. Adjusted 2: covariates were age, sex, Charlson Comorbidity Index, type of surgery, type of anaesthesia; *N*=821. Sensitivity 1: covariates were age, sex, education (ISCED), Charlson Comorbidity Index; *N*=759. Sensitivity 2: covariates were age, sex, imputed Geriatric Depression Scale, Charlson Comorbidity Index, frailty (prefrail, frail, robust), and the sum of taken drugs; *N*=720. AUROC, area under the receiver operating characteristic curve; CI, confidence interval.

	Odds ratio	95% CI	AUROC	95% CI
Unadjusted	1.06	1.02–1.09	0.60	0.55–0.64
Adjusted 1	1.04	1.01–1.08	0.63	0.58–0.67
Adjusted 2	1.05	1.01–1.09	0.67	0.63–0.72
Sensitivity 1	1.05	1.01–1.09	0.66	0.61–0.70
Sensitivity 2	1.03	0.99–1.07	0.65	0.60–0.70

### Sensitivity analyses

We first performed logistic regressions including covariates that were used by the replicated studies. When including age, sex, education (ISCED), Charlson Comorbidity Index as was done by Rudolph and colleagues,^11^ our analysis comprised 759 complete cases (postoperative delirium in *N*=137). The OR for a 10-s increment in the TMT-B was 1.05 ([95% CI 1.01–1.09], *P*=0.02). The calculated AUROC was 0.66 ([95% CI 0.61–0.70], *P*<0.0001).

To replicate Mychajliw and colleagues,^10^ we used the covariates age, sex, imputed GDS, Charlson Comorbidity Index, frailty (prefrail, frail, robust), and the sum of taken drugs. The BioCog variable frailty turned out to have 223 values missing. We listed the number of missing values for each covariate in Supplementary Table S1. After omitting frailty as a covariate, 720 complete cases were included (postoperative delirium in *N*=135). TMT-B performance was not significantly associated with postoperative delirium incidence (OR per 10-s increment 1.03 [95% CI 0.999–1.07], *P*=0.10] with an AUROC of 0.65 ([95% CI 0.60–0.70], *P*<0.0001). Multicollinearity could be ruled out for all the sensitivity analyses.

### Additional analyses

For the third sensitivity analysis, we identified 21 potential outliers according to their TMT-B scores (Supplementary Table S2). Subsequently, we excluded these outliers and re-ran the logistic regression analysis, taking postoperative delirium as a dependent variable. The TMT-B yielded an OR of 1.07 per 10-s that was required to finish the test ([95% CI 1.03–1.07], *P*<0.001). The AUROC was 0.60 ([95% CI 0.55–0.65], *P*<0.001).

For 88 patients TMT-B data were either missing or considered impaired. Missing or impaired test results were also associated with the onset of postoperative delirium (OR 2.74 [95% CI 1.71–4.35], *P*<0.001). The simple logistic regression model resulted in an AUROC of 0.55 ([95% CI 0.50–0.60], *P*=0.03). Of *n*=31 impaired results, *n*=24 were suitable for a *post hoc* worst-case imputation, where a test result of 301 s was used. The OR for the TMT-B remained unchanged (OR per second increment; 1.06 [95% CI 1.04–1.09], *P*<0.001). The AUROC was 0.62 ([95% CI 0.56–0.66], *P*<0.001).

We tested the cut-off of 154 s provided by Greene and colleagues.^8^ It yielded a sensitivity of 24.5%, whereas the specificity was 82.8%.

## Discussion

We consider this replication study a success. We were able to observe an association between preoperative TMT-B results and the onset of postoperative delirium, which replicated most of the previous studies that also demonstrated a significant association. More specifically, the longer it took patients to complete the TMT-B, the higher the odds they would develop postoperative delirium. For each additional 10 s required to finish the TMT-B, the probability for postoperative delirium increased by 6%. However, the predictive value of the preoperative TMT-B performance was low in discriminating those who would develop postoperative delirium from those who would not develop postoperative delirium.

Four previous studies analysed the association between preoperative TMT-B test performance and the incidence of delirium. Three out of four reported an association of TMT-B with delirium. None of these studies described the circumstances of how cognitive data were obtained or considered plausible. The quality of reporting, statistical rigor, and choice of methodology varied between studies. Future studies are advised to at least report the test procedures and scoring in more detail and preferably standardise these between studies according to published test manuals.

Rudolph and colleagues^11^incorporated the TMT-B into a composite executive functioning score and, therefore, the TMT-B did not represent their primary focus of the analysis. For analysis reasons, they set an arbitrary threshold of 0.5 standard deviation below the mean that classified TMT-B test scores impaired. Unlike Rudolph and colleagues,^11^ when using comparable covariates as used in the original study, we found an association between TMT-B with the odds for postoperative delirium.

Whereas patients received a TMT-B testing at baseline, Mychajliw and colleagues^10^ only interviewed them retrospectively about symptoms that may possibly be indicative for past episodes of delirium. Furthermore, only 69% of the recorded delirium cases were attributed to surgery as the primary cause. Taken together with the arbitrary selection of covariates, it is not surprising that we were unable to replicate their results. We only included this study to provide a complete overview of all studies that associate the TMT-B with delirium. However, it remains questionable whether their approach could be of much use for the prediction of postoperative delirium.

The study of Greene and colleagues^8^ was designed to provide only preliminary results and consisted of 91% male participants, introducing a sex bias. Furthermore, delirium status was only assessed once daily and only for the duration of three consecutive days after surgery. It remains unclear whether screening started on the day of surgery. The TMT-B was chosen as the predictor cognitive test after univariable analyses of different cognitive tests, but this approach is generally not advised.^24^

Only Lindroth and colleagues^9^ solely focused on the TMT-B without performing an initial exploratory analysis into other cognitive tests. Their study primarily investigated delirium severity, whereas the relation of the TMT-B to postoperative delirium served as an exploratory outcome.

The test threshold used during analysis proposed by Greene and colleagues^8^ for predicting postoperative delirium, yielded an insufficient sensitivity and specificity in our replication sample. Before the preoperative TMT-B could be considered as a prediction tool for postoperative delirium, reliable performance cut-offs or standardised scores must be developed. This requires normative data derived from a much larger cohort. Despite its insufficient predictive performance as a stand-alone test, the TMT could be used in prediction algorithms alone or together with other easy to obtain scores. For instance, Greene and colleagues^8^ added the GDS and Lindroth and colleagues^9^ the NSQIP-SC score to the TMT-B. The AUROC of the latter model was 0.81, whereas the extent of the TMT-B’s effect remains unclear. However, it might be a promising prediction approach and should be considered in future studies.

Apart from Rudolph and colleagues,^11^ the replicated studies in unison interpret the TMT-B results as a measure of executive functioning. Indeed, executive functioning was observed to be important in emergence from anaesthesia and was associated with postoperative delirium.^11^^,^^25^^,^^26^ However, executive function encompasses an array of different functions of which cognitive flexibility is one element as assessed by the TMT-B. The TMT-B as a single test cannot fully reflect executive functioning in its complexity.^7^ The TMT is dependent on other cognitive domains as well, including visual attention, working memory, and psychomotor performance.^6^^,^^27^ To examine executive functioning more extensively, cognitive test batteries are probably more suitable. These may help to assess a larger range of cognitive domains, which are commonly summarised under the term executive function. Nonetheless, the TMT-B might be indicative for the domains important for the overall cognitive performance of older adults and because of its ease of application, may be more suitable as a quick screening tool. The Mini-Cog battery might represent another promising approach to use preoperative cognitive performance as a predictor of postoperative delirium as it was shown to be associated with postoperative delirium.^28^ The Mini-Cog comprises clock-drawing, memory tasks and can be finished within minutes. Of note, administering the TMT-B can take up to 5 min, while only resulting in an impaired test result. It remains questionable whether this approach is feasible or desirable in a clinical setting. Future studies should further investigate the TMT-B’s performance in prediction algorithms, possibly in comparison to or combination with larger cognitive test batteries.

## Strengths

The BioCog study combines a variety of methodological strengths that make it suitable for the replication of studies of perioperative neurocognitive disorders. BioCog has a large-scale design with two recruitment centres, resulting in a considerable sample size eligible for replication studies. Postoperative delirium screening was in line with DSM 5 and corresponding guidelines.^19^ Unlike in many other studies, the postoperative delirium assessment was carried out twice a day starting in the post-surgery recovery room. Thereby, the screening intervals accounted for the fluctuating nature of delirium.^19^ The TMT-B testing was conducted according to the test manual and administered by trained personnel.^20^ Moreover, the stability of cognitive test results has been ascertained by using the data of a non-surgical control group.^21^ Taken together, these strengths show that our replication study has the potential to strengthen the conclusions of previous studies.

## Limitations

This is a secondary replication study using existing data. We would like to encourage other researchers in the field to incorporate aspects of study replication to future studies at the design stage. Another limitation of our study concerns missing data. It potentially obfuscates the results of the primary analysis and the sensitivity analyses. Although there are many similarities to the BioCog study, we were unable to directly replicate the model of Lindroth and colleagues^9^ including covariates as we were lacking the NSQIP-SC variable. Hence, we are unable to draw conclusions on the discriminatory performance of this model in a different and bigger sample size. Because of missing data, we only partly used the same variables as Mychajliw and colleagues.^10^ However, the initial study itself calculated means for the Charlson Comorbidity Index and frailty because of missing data.

As depicted in the sensitivity analyses, impaired TMT-B performance was also associated with the development of postoperative delirium. We chose to perform a *post hoc* worst-case imputation for impaired test performances.

We did not integrate error values into our investigation. However, error values potentially imply important information and, therefore, might be included to future TMT-B related analyses.

## Conclusion

We were able to replicate previous studies in the finding that poorer TMT-B test performance was associated with increased odds of postoperative delirium. We conclude that this effect replicates the effect found by three prior studies. However, the discriminatory performance of TMT-B scores was insufficient. Accordingly, we do not recommend the use of the TMT-B as a stand-alone predictive tool for postoperative delirium in clinical practice. Nonetheless, our findings may help to elucidate the association of different cognitive domains as measured by the TMT-B performance with the vulnerability for postoperative delirium.

## Authors’ contributions

Conceptualisation, software, visualisation, writing—original draft: MF

Data curation: MF, IF, FB, MH

Formal analysis, investigation: MF

Methodology: MF, DSV

Validation: MF, IF, FB

Writing—review and editing: all authors

Supervision: IF, DSV, TP, GW, NZ

Funding acquisition: TP, AJCS, CDS, GW

Project administration, resources: AJCS, CDS, GW

## Funding

The project ‘Biomarker Development for Postoperative Cognitive Impairment in the Elderly’ (BioCog) was supported by the European Community’s FP7 (602461).

## Appendix

### Research Consortium

The BioCog Consortium also consists of Alissa Wolf, Anika Müller, Daniel Hadzidiakos, Fatima Yürek, Gunnar Lachmann, Kwaku Ofosu, Rudolf Mörgeli (all Charité–Universitätsmedizin Berlin), Jürgen Gallinat, Simone Kühn (all University Medical Center Hamburg), Edwin van Dellen, Ilse Kant, Jeroen de Bresser, Jeroen Hendrikse, Simone van Montfort (all University Medical Center Utrecht), David Menon, Emmanuel Stamatakis, Jacobus Preller, Laura Moreno-López, Stefan Winzeck (all University of Cambridge), Daniela Melillo, Diana Boraschi, Giacomo Della Camera, Paola Italiani (all National Research Council Napoli), Reinhard Schneider, Roland Krause (all University of Luxembourg), Karsten Heidtke, Peter Nürnberg (all ATLAS Biolabs GmBH, Berlin), Anja Helmschrodt, Axel Böcher, Bettina Hafen, Franz Paul Armbruster, Ina Diehl, Jana Ruppert, Katarina Hartmann, Marion Kronabel, Marius Weyer, Thomas Bernd Dschietzig (all Immundiagnostik AG, Bensheim), Malte Pietzsch, Simon Weber (all Cellogic GmbH, Berlin), Bernd Ittermann, and Ariane Fillmer (all Physikalisch-Technische Bundesanstalt, Berlin).
