# Supplementary material for: Trail making test B in postoperative delirium: a replication study
Source: BJA Open. 2023 Nov 3;8:100239. doi: 10.1016/j.bjao.2023.100239 (PMC10633257; doi:10.1016/j.bjao.2023.100239)
Supplement: Multimedia component 1 [file mmc1.docx]

## Supplemental Figure 1: Receiver Operator characteristics curve


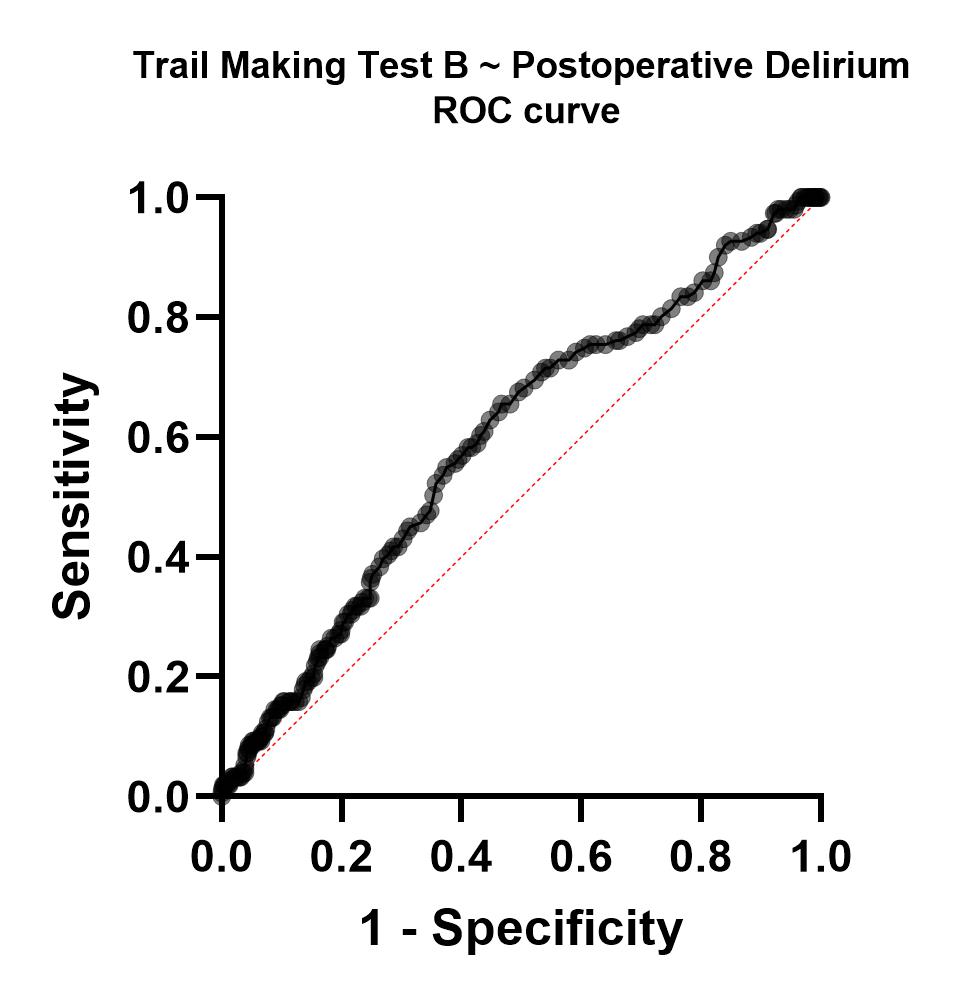


Note: Receiver Operator Characteristics curve showing the performance of preoperative Trail Making Test B scores predicting postoperative delirium. The area under the curve is 0.60 [(95% CI 0.55 – 0.64) p< 0.001] for n = 841.

## Supplemental Table 1: Summary of Missing Data

| Variable | Number of Missing Data |
| --- | --- |
| Age | 0 |
| Sex | 0 |
| ISCED | 77 |
| Preoperative Charlson Comorbidity Index | 4 |
| GDS | 117 |
| Frailty | 223 |
| Sum of taken drugs | 91 |

|  | Trail Making Test B score (in seconds) |
| --- | --- |
| 1 | 288 |
| 2 | 253 |
| 3 | 256 |
| 4 | 268 |
| 5 | 245 |
| 6 | 248 |
| 7 | 250 |
| 8 | 270 |
| 9 | 268 |
| 10 | 291 |
| 11 | 252 |
| 12 | 284 |
| 13 | 256 |
| 14 | 294 |
| 15 | 298 |
| 16 | 264 |
| 17 | 260 |
| 18 | 259 |
| 19 | 270 |
| 20 | 271 |
| 21 | 295 |

## Supplemental Table 2: Outlier and respective Trail Making Test B scores
